# Medical Student Experiences of Engaging in a Psychological Flexibility Skill Training App for Burnout and Well-being: Pilot Feasibility Study

**DOI:** 10.2196/43263

**Published:** 2023-01-10

**Authors:** Elizabeth Ditton, Brendon Knott, Nicolette Hodyl, Graeme Horton, Frederick Rohan Walker, Michael Nilsson

**Affiliations:** 1 Centre for Rehab Innovations University of Newcastle Callaghan Australia; 2 Hunter Medical Research Institute New Lambton Heights Australia; 3 College of Health, Medicine and Wellbeing University of Newcastle Callaghan Australia; 4 Contextual Interventions Newcastle Australia; 5 New South Wales Regional Health Partners Newcastle Australia; 6 Lee Kong Chian School of Medicine Nanyang Technological University Singaport Singapore

**Keywords:** medical students, burnout prevention, app, feasibility, intervention engagement, psychological flexibility, acceptance and commitment therapy, mobile phone

## Abstract

**Background:**

Medical students are at higher risk of burnout than the general population. Interventions that facilitate adaptive coping behaviors (eg, Psychological Flexibility) in the context of inherent stressors associated with medical training could mitigate burnout risk and improve well-being. Delivering these interventions using smartphone apps offers advantages such as accessibility, scalability, mitigation of time and stigma barriers, and facilitation of individual tailoring (*individualization*). There is a need for feasibility trials with medical students in this emerging field. Formal evaluations of user experiences of app-based psychological skill training are required to identify barriers to and facilitators of engagement and optimize intervention development before implementation in efficacy trials and real-world settings.

**Objective:**

This study aimed to assess the feasibility of delivering an individualized Psychological Flexibility skill training intervention (Acceptance and Commitment Training [ACTraining]) to medical students using an app-based delivery format. We further aimed to explore how formal evaluation of user experiences might inform and guide the development of this app before implementation in an efficacy trial and future research involving app-delivered psychological skill training for medical students.

**Methods:**

This single-arm study was an early-phase feasibility trial of a stand-alone ACTraining app conducted with a sample of Australian medical students (n=11). We collected app usability and user experience data across a broad range of domains (eg, perceived helpfulness and relevance, learning experiences, and self-efficacy) using self-report questionnaires (quantitative and qualitative) and behavioral engagement outcomes.

**Results:**

Behavioral engagement data demonstrated that the app delivered the assessment procedures and individualized ACTraining intervention to medical students as intended. The subjective feedback provided by students who actively engaged with the app was generally positive across several indicators, including usability, perceived relevance and helpfulness, accessibility, maintenance of privacy, and opportunity for self-reflection. Disengagement from the app was an identified challenge throughout the trial. Participant feedback identified several factors that may have affected engagement, such as time, expectations regarding app interface functioning, and individual differences in confidence and self-efficacy when implementing skills.

**Conclusions:**

This study reports user experience data that have been largely absent from the literature on digital psychological interventions for medical students. Our findings demonstrate the preliminary feasibility of an app-delivered ACTraining intervention for medical student well-being and burnout and support the value of future assessment of the efficacy of this approach with larger samples. We consider subjective feedback from medical students in relation to observed engagement and propose how this information might be used to inform the development of this app and future research in this nascent field.

## Introduction

### Medical Student Stress and Psychological Health

The stress of medical training can have adverse effects on students’ psychological health [1-3]. Worldwide, medical students are at an elevated risk of psychological ill health compared with the general population [1], demonstrating high prevalence of burnout (44.2% [4]), depression (27.2% [2]), and suicidal ideation (11.1% [2]). Burnout risk increases as training progresses [5] and can persist throughout a physician’s professional life [6]. The detrimental consequences of burnout and psychological ill health among medical students and physicians are well documented and include poor physical health [7], diminished life satisfaction [6], and disruption to health care service delivery (eg, impaired performance [8] and reduced empathy [5] and work engagement [9]).

### Adaptive Psychological Skill Training

Medical educators report increasing concern regarding the psychological health of students [10] and recognize the need for early intervention strategies that can prevent burnout rather than treat it after it has already emerged [10-12]. In addition to organizational approaches that mitigate modifiable external stressors (eg, improving learning environments and addressing student mistreatment [13]), there is support for initiatives that build individual psychological and behavioral resources for coping with unmodifiable stressors encountered during medical education and practice [10,12,14,15] (eg, academic pressures and exposure to illness and death [5,6]). This intervention strategy has the potential to buffer medical students against burnout and psychological ill health by facilitating adaptive responses to stressful experiences [6,15,16]. Beyond simply preventing psychological ill health, adaptive psychological skill training can improve well-being [17,18]. Well-being is a state of personal thriving and vitality that is an essential component of complete psychological health [19,20] and a key outcome of interest to medical educators [10]. There is support for the acceptability of adaptive psychological skill-building interventions among medical students [3] and physicians experiencing burnout, with the latter having rated such training as being as important as learning clinical skills [21].

### App-Delivered Interventions

The increased interest in medical student psychological health has resulted in a growing number of studies evaluating individual skill-building interventions [3]. A notable gap in this emerging field is the absence of studies evaluating the delivery of such interventions using stand-alone smartphone apps [1,3]. Although the preliminary feasibility of app-delivered interventions (eg, mindfulness) has been demonstrated among small samples of medical residents [22,23], there are no studies evaluating feasibility among medical students. Medical students use smartphones frequently and are accustomed to engaging with technology [1,24,25] and, thus, app-delivered interventions have the potential to be accessible [1,26] and scalable [12]. Apps could facilitate medical students’ access to psychological skill training anytime and anywhere [27], which is particularly important when the availability of psychological health care personnel is limited or face-to-face delivery is not practical. App-delivered interventions may also offset known barriers to medical student engagement in psychological interventions by maintaining anonymity and privacy for students concerned about mental health stigma [1,6] and delivering brief intervention components that minimize participation-related time pressures [28]. Finally, app-delivered interventions provide a potential solution to the problem of heterogeneity and variation in individual responses to psychological interventions [29] by facilitating the tailoring of training to individual medical student needs (ie, *individualization*) [27]. Individualized psychological apps have demonstrated greater effectiveness than nonindividualized apps with respect to well-being outcomes among heterogeneous university student samples [27].

Despite the potential advantages of app-delivered interventions for medical student psychological health, there are several challenges that could affect the feasibility of this strategy. Maintaining individual participation in psychological health apps is a known problem generally, with real-world user engagement diminishing substantially within 2 to 4 weeks of sign-up [30]. Engagement can be affected by a range of usability and acceptability factors, including the quality and functionality of the interface [1,31], training content relevance [26], the context in which the intervention is experienced [31], time burden [22], and user experiences [1] (eg, affective responses, perceived effectiveness, and self-efficacy [31]).

Given the infancy of this emerging field and intervention delivery mode, feasibility assessment is an important precursor to successful implementation in larger efficacy trials [32]. There is a need for studies that formally evaluate medical students’ subjective experiences of engaging in app-delivered psychological interventions as well as their perceptions of interface usability [1,33]. The absence of this information from the literature has been noted as a hindrance to progressing digitally delivered psychological skill interventions beyond the prototype phase to effective, fully developed products that could be successfully implemented in medical education settings [1]. The collection of these data can provide information about how psychological skill training apps are experienced in real-world contexts among medical student end users [32,34], unexpected methodological issues and barriers [32], and factors that may affect engagement [34,35].

### This Study

This study is a small-scale feasibility assessment of an adaptive psychological skill training app for medical student burnout prevention and well-being using an evidence-informed behavioral model (Psychological Flexibility).

#### Intervention Model: Psychological Flexibility

There are no standardized psychological skill training interventions available for medical student burnout and psychological well-being [3]. However, Psychological Flexibility is an adaptive behavioral skill set that has been shown to protect against adverse mental health outcomes in stressful situations [18,36-41] and is emerging as a promising intervention target [17,18,37,42]. The model encompasses 6 modifiable behavioral flexibility (and corresponding inflexibility) processes: present-moment awareness (nonawareness), experiential acceptance (avoidance), cognitive defusion (fusion), self-as-context (self-as-content), contact with values (noncontact with values), and committed action toward values (inaction) [43]. When exposed to stressful situations, individuals who are high in Psychological *Inflexibility* tend to respond in ways that are rigidly driven by their internal experiences (eg, fusion with unhelpful thoughts, rules, or self-stories, and avoidance of emotional discomfort). This can increase their risk of adverse psychological outcomes by restricting coping repertoires, disrupting engagement in personally meaningful behaviors, and exacerbating distress [36,44,45]. Conversely, as psychologically flexible individuals are more able to adopt an open and nonevaluative stance toward internal experiences, they are less likely to interpret emotionally uncomfortable stressors as threatening to their well-being [36]. By focusing less on avoiding or reducing discomfort and more on connecting with and accepting their direct experiences, psychologically flexible individuals are able to recognize a broader range of situational opportunities available to them when responding to stressful conditions [40,46]. This facilitates purposeful action toward personally held values and related goals [43]. Early evidence supports the potential relevance of Psychological Flexibility skills to medical students, who are less likely to experience burnout when they adopt experiential acceptance [6,47] and engage in values-driven actions [48]. Conversely, low Psychological Flexibility among medical students is associated with an elevated risk of burnout [49], reduced life satisfaction, and greater personal distress when seeing others in harm [50].

#### Acceptance and Commitment Training

Psychological Flexibility skills can be developed and strengthened using Acceptance and Commitment Training (ACTraining) [43]. ACTraining interventions implemented in medical education settings have demonstrated beneficial impacts such as reduced burnout among distressed medical students [51] and improved well-being and diminished psychological distress among female medical students [52]. ACTraining is well suited to an app-based format as it teaches Psychological Flexibility skills using metaphors and experiential activities that can be delivered as brief and practical training components [27]. By facilitating opportunities to practice during the course of medical students’ everyday lives, app-delivered ACTraining interventions could strengthen and generalize Psychological Flexibility skills [27]. Furthermore, as ACTraining components have functional links with their corresponding Psychological Flexibility processes [43,45,53], interventions can be *individualized* within an app by identifying which skill a student requires the most assistance with in a particular moment and aligning the delivery of targeted components with these needs [27]. This is important given the heterogeneity of Psychological Flexibility skill profiles between individuals and the fact that the 6 processes can vary independently of one another [44,54,55], suggesting that medical students are likely to require training in different skills at different times and in different situations [44].

At the content level, ACTraining offers potential feasibility benefits with respect to its acceptability and relevance to medical students. ACTraining adopts a normalizing stance toward uncomfortable internal responses to stressors (ie, thoughts, emotions, and physical sensations) [43,56], providing opportunities to learn adaptive behavioral skills in a nonstigmatizing way. Furthermore, as ACTraining encourages action driven by awareness of an individual’s unique experiences, values, and goals [43], students can learn to apply the skills in ways that are personally relevant. However, aspects of ACTraining (eg, self-reflection and bringing attention to uncomfortable thoughts and emotions) may be challenging when experienced in a self-directed app-based context without guided support [26]. Evaluating medical students’ subjective experiences of engaging with ACTraining content is an important aspect of feasibility assessment, particularly given the potential impact on app engagement [26]. To our knowledge, no feasibility data of this nature have been reported previously.

#### Study Aims and Objectives

We developed an individualized Psychological Flexibility skill training app (using an ACTraining intervention approach) for medical student burnout and well-being. The aims of this study were to assess the feasibility of delivering an ACTraining intervention to medical students using an app-based delivery format, and explore how formal evaluation of subjective user experiences might inform and guide the development of this app before implementation in a planned efficacy trial as well as future research regarding psychological skill training apps for medical students. This study was an early implementation trial conducted with a small sample of Australian medical students who were interested in engaging in an app-based burnout prevention and well-being intervention. We collected behavioral engagement data and detailed student feedback regarding the ACTraining intervention content, usability of the app interface, and experiences of engaging with the intervention across a range of acceptability domains (eg, affective responses, perceived learning, self-efficacy, and engagement barriers and facilitators).

## Methods

### Study Design

This study was a single-arm feasibility trial of a 2-stage individualized ACTraining app.

### Participants, Recruitment, and Study Setting

We aimed to recruit approximately 10 to 15 participants, consistent with sample size recommendations for feasibility studies [35]. As the app had not been trialed previously, we limited the number of participants to allow for a smaller-scale, detailed end-user evaluation before broader implementation in a planned larger efficacy trial [57]. Recruitment occurred over a 3-week period in June 2021.

The sampling frame was all students enrolled in year 3 of the Joint Medical Program at the University of Newcastle and University of New England (Australia) during semester 1 of 2021 (N=170). Year 3 students were selected for pragmatic reasons (eg, timing of the study in relation to academic demands and the impact of the COVID-19 pandemic), as recommended by the University of Newcastle head of medical student well-being (GH). Students were eligible for inclusion if they had regular access to a reliable internet connection and an electronic device compatible with app use (smartphone or tablet). Although the ability to understand English was a requirement for using the app, this was not stated explicitly as all members of the sampling frame met this criterion. There were no ineligibility criteria.

**Figure 1:**
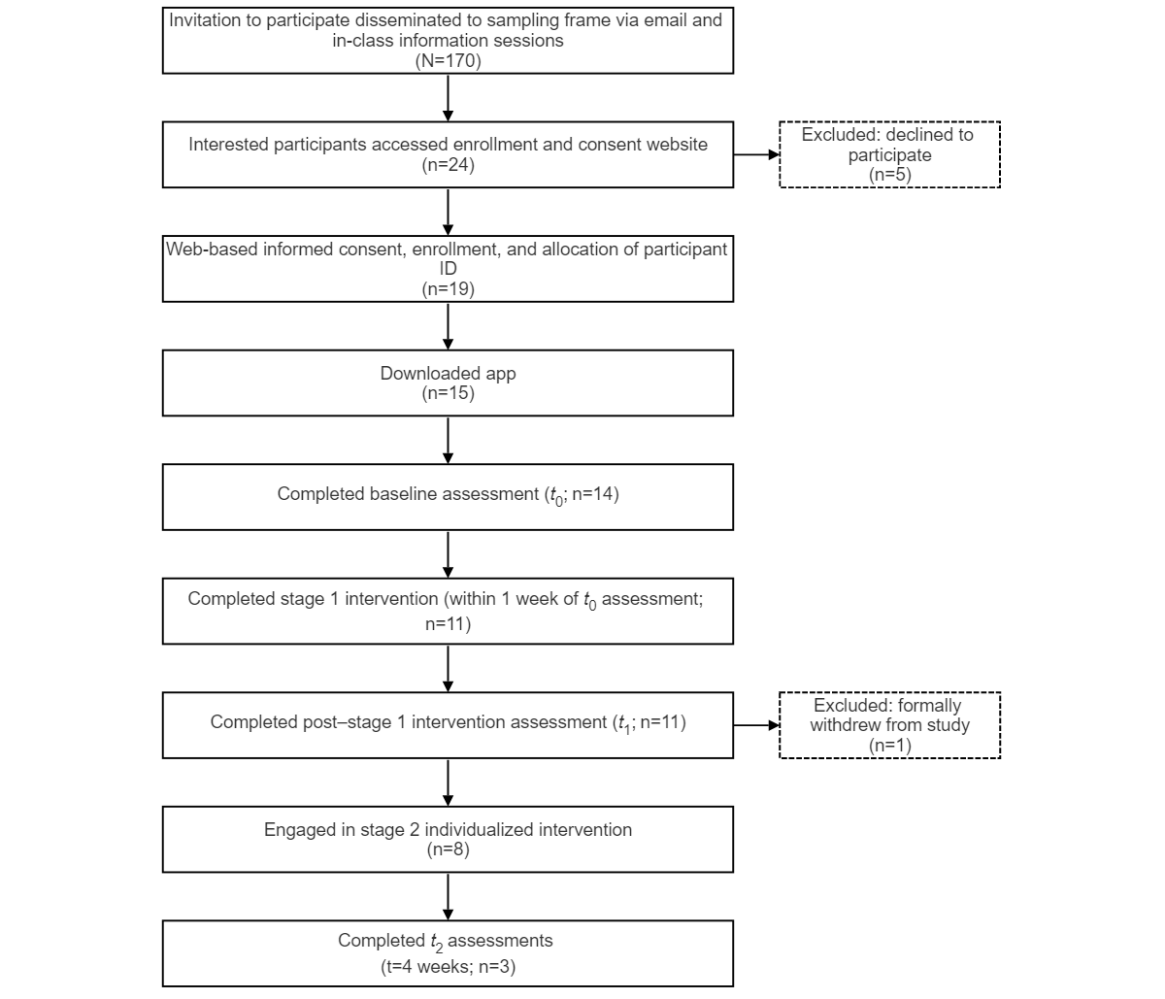
Participant flow diagram.

All aspects of the study were implemented on the web. A digital invitation was sent from a Joint Medical Program administrative account to the students’ university email accounts, and a follow-up invitation was sent a few days before the final enrollment date. Students also received a verbal invitation at the end of a web-based class delivered by a member of the research team. Participation in the study was voluntary. Students were given a URL and QR code that allowed them to access the enrollment website (hosted on a secure web-based survey and database platform, REDCap [Research Electronic Data Capture; Vanderbilt University]) that provided information about the study and allowed students to enroll using an e-consent process. After completing a brief demographic questionnaire, consented students were allocated a unique (4-digit) participant ID and provided links to download the app via the App Store (Apple) and Play Store (Android). After downloading the app, students created an account. All further assessment, data collection, and intervention procedures were conducted via the app, which students accessed independently during the study period. Self-report outcomes were measured at 3 time points: baseline (*t*_0_), following stage 1 of the intervention (*t*_1_), and following stage 2 of the intervention (*t*_2_). Owing to the time commitment involved in the study, an Aus $30 (US $20.43) gift voucher was offered to participants who completed the intervention and self-report outcome measures. Figure 1 shows the participant flow diagram.

#### Ethical Considerations

Ethics approval for this study was granted by the University of Newcastle Human Research Ethics Committee on January 21, 2021 (approval ID: H-2020-0311) and ratified by the University of New England Human Research Ethics Committee on February 11, 2021. To ensure the privacy and anonymity of participant data, 2 password-protected databases were used to store personal and demographic information (in an identifiable *Participant Information Database*) separately from the outcome data (stored in an anonymous *Study Database*). Outcome data were deidentified, with participant ID used as a linkage key. Only the lead author had access to the Participant Information Database. Members of the research team did not have access to identifying participant information. To protect privacy while using the app, students input their unique participant ID as their username when creating and accessing their accounts.

Before providing consent, students were informed that (1) data collected during the study would be anonymous and retained and stored for a minimum period of 5 years from completion of the research, in accordance with the University of Newcastle research data management policies; (2) deidentified data would be analyzed, summarized, and presented in various formats (eg, peer-reviewed journal publications); (3) nonidentifiable data may be used for future ethically approved research; and (4) if students elected to withdraw from the study, data provided up to the point of withdrawal would be retained and included in the study in a deidentified and anonymized form.

### Intervention

#### Overview

The intervention involved the delivery of individualized ACTraining via an app (“BiSi: Build it. Sustain it.”) developed by clinical psychologists (ED and BK). Existing Psychological Flexibility training activities and concepts were adapted to suit the context and target participant group. The app was created in a low-code platform (Cogniss) that facilitated the delivery of the outcome measures and intervention and the collection of study data (including participants’ responses to outcome measures and use data). Intervention content was delivered in audio format with accompanying images and some supporting written text. The ACTraining intervention was delivered in 2 stages. Further information regarding the *Build it. Sustain it.* app and intervention protocol has been published elsewhere [57].

#### Stage 1: Introductory Module

During stage 1, participants used the app to complete an introductory module that provided a conceptual framework from which to understand the full Psychological Flexibility skill set. The module was divided into 7 sections (<10 min each), which were presented in a fixed order and took approximately 1 hour to complete. Progress was saved at the end of each section, allowing participants to pause and resume the module if they wished to complete it over multiple sittings. Section 1 content included an introduction to burnout (definition, recognizing the signs, and normalizing and destigmatizing) and well-being (definition and importance of choosing behaviors that support career sustainability). Sections 2 to 7 provided education about each Psychological Flexibility skill set (see the screenshot in Multimedia Appendix 1), including the role of psychologically inflexible behaviors in burnout and psychological distress and how flexibility processes may facilitate more adaptive responses and psychologically healthy outcomes. Although the focus of stage 1 was predominantly conceptual, each Psychological Flexibility process was accompanied by at least one skill practice activity (including personal value identification, metaphors, mindfulness practice, awareness practice, thought defusion, perspective-shifting techniques, and behavioral activities). Participants were given 1 week to complete stage 1.

#### Stage 2: Individualized Skill Training

Stage 2 was designed to provide students with on-demand access to a library of short (3-8 min each) practical Psychological Flexibility skill activities (20-25 activities per process), which were targeted to their specific training needs each time they accessed the app. Completion of stage 1 unlocked access to the stage 2 dashboard (see the screenshot in Multimedia Appendix 2), which students could access at any time. Although there was no requirement to complete a specific number of activities, participants were sent daily reminder emails and encouraged to practice regularly to optimize skill learning. Activities focused on normalizing challenging internal experiences, strengthening present-moment awareness, recognizing the influence of internal experiences (including thoughts and emotions) on choices and actions, learning to alter unworkable responses to internal experiences, setting values-based goals, and developing more flexible and value-aligned behavioral repertoires [43,45,58]. Table 1 provides a summary of the aims and an example activity for each Psychological Flexibility skill set.

**Table 1:** Aims and example activities for each Psychological Flexibility skill set.

Skill set	Aim	Example activity
Present-moment awareness	Teach students to bring purposeful awareness to their present-moment experiences and recognize how these experiences influence their choices (in everyday life and stressful situations)	Guided mindfulness activity in which students practice attending to specific aspects of their present-moment experience (eg, sounds, physical sensations, thoughts, emotions, and breathing)
Defusion	Teach students to distinguish between their direct experiences and their thoughts about their experiences and respond more flexibly to the latter by changing the contexts in which they experience their thoughts	Students practice responding flexibly to a thought by choosing an action that contradicts a thought they are having (eg, they are asked to think “I can’t lift my arm” and to also lift their arm).
Self-as-context (perspective taking)	Teach students to recognize that their thoughts and emotions do not define their identity but are transient aspects of their experience, which they can observe from a more stable and constant sense of “self”	Students identify a “self-story” that causes distress or limits their response options in certain situations and practice choosing actions that expand their behavioral repertoires outside of these stories.
Acceptance (willingness)	Teach students to identify unhelpful avoidance behaviors and practice open, flexible, and adaptive ways of responding to internal discomfort	Students practice observing an uncomfortable sensation (eg, an urge or an itch) with openness, curiosity, and willingness (eg, notice the components of the sensation and allow them to come and go without buying into evaluations of the sensations or attempting to alter them).
Values	Teach students to identify their own personally held values and notice how connection or disconnection with these values can contribute to well-being or burnout experiences, respectively	Students choose one of their own personally held values and briefly reflect on how their behaviors have aligned with that value during the day.
Committed action	Teach students to choose effective and purposeful behaviors that align with personally held values even when internal discomfort is present; use direct experiences to recognize the difference between well-being–oriented action and inflexible persistence that could increase burnout risk	Students select a personally held value that feels most important to them in that moment and commit to taking one small and achievable action toward that value during the day.

Individual tailoring of Psychological Flexibility skill activity delivery was implemented using a method adapted from a similar previous study [27]. At each app log-in, participants responded to a single-item screening question: *which of the following are you having the most difficulty with today?* (see the screenshot in Multimedia Appendix 3)*.* Each response option corresponded to 1 of the 6 Psychological Flexibility processes (Textbox 1). After selecting a response, participants were directed to a dashboard that provided access to all skill activities available for the Psychological Flexibility process they had identified as having the most difficulty with on that occasion (presented in a list). Students were then able to practice one of the available skill activities, either by choosing from the list or by allowing the app to make a random selection for them (see the screenshot in Multimedia Appendix 4). Upon completion of an activity, participants had the option to complete another activity within the same Psychological Flexibility skill set. These steps were repeated until the participants elected to discontinue. Participants had access to stage 2 for 3 weeks.

**Stage 2 individualization screening question options and corresponding Psychological Flexibility processes.**

- Struggling with your feelings: acceptance (willingness)
- Unable to do what matters to you: committed action
- Stuck in your thoughts: defusion
- Stuck in autopilot or struggling to stay in the present moment: present-moment awareness
- Disconnected from a sense of meaning or purpose: values
- Stuck in stories about who you are or who you should be: self-as-context (perspective taking)

#### Engagement

We integrated design elements aimed at enhancing medical students’ engagement with the app-based intervention. In addition to individualization [27], this included the provision of daily reminder emails [59], offering a variety of training activities for each skill set [27,60], and providing opportunities to engage in self-reflection while engaging in the educational and skill practice components of the app [60].

### Psychological Outcome Measures

#### Overview

We administered self-report psychological outcome measures (Table 2) at *t*_0_ only. The purpose of administering these outcome measures in this study was to evaluate the feasibility of using the app delivery method to assess psychological outcomes of interest in the planned efficacy trial and explore the potential assessment burden to participants. Psychological outcome data were also used to describe the baseline psychological characteristics of this feasibility trial sample.

**Table 2:** Summary of outcome measure characteristics and internal consistency (Cronbach *α*) for this sample.

Outcome measure			Items, N		Measure characteristics		Cronbach *α*
**Psychological outcomes**							
	Burnout—MBI-GS(S)^a^ [61]	16		Validity [62] and reliability have been demonstrated among medical students [63]. Respondents rate the frequency of each burnout experience using a 7-point Likert scale. Provides summed total scores for each burnout factor; higher E^b^ and C^c^ and lower AE^d^ scores reflect a higher frequency of burnout experiences.		.84	
	Well-being—MHC-SF^e^ [19]	14		Validity [19] and reliability (including internal consistency and test-retest reliability) [64] have been demonstrated. Respondents rate the frequency of well-being experiences during the previous month using a 6-point Likert scale. Provides average item scores for 3 well-being factors: EWB^f^, SWB^g^, and PWB^h^. Higher scores reflect a higher well-being.		.91	
	Psychological Flexibility and Inflexibility—MPFI-SF^i^ [54]	24		This scale has demonstrated validity (including convergent, predictive, and discriminant validity between PF^j^ and PI^k^ scales [55]) and reliability (including internal consistency [65] and responsiveness to change over time [54]). Respondents rate the frequency of PF and PI experiences during the past 2 weeks using a 6-point Likert scale. Provides global PF and global PI composite scores. High PF scores reflect higher flexibility, and higher PI scores reflect higher inflexibility.		.89	
	Depression, anxiety, and stress—DASS-21^l^ [66]	21		Validity and reliability have been demonstrated [66,67]. Respondents rate the frequency of depression, anxiety, and stress symptoms experienced during the past week using a 4-point Likert scale. Provides subscale scores estimating the severity of depression, anxiety, and stress symptoms [66] and a total score reflecting general negative affectivity or psychological distress [67].		.91	
**Feasibility outcomes**							
	App usability—SUS^m^ [27]	10		Validity and reliability have been demonstrated [27]. Respondents rate the degree to which they agree with usability statements about the app using a 5-point Likert scale (1=“strongly disagree” to 5=“strongly agree”). Provides a total score ranging from 0 to 100. Higher scores reflect higher usability (85-100=excellent, 73-84=good, 62-73=low marginal, and <70=needs to be reviewed and improved) [68]. In accordance with recommendations by other authors, we amended item wording to suit our intervention context and audience (eg, “system” changed to “App”) [69].		.81	
	User Experience Assessment part 1	32		Respondents rate their level of agreement with each statement about experiences of engaging with the intervention app using a 5-point Likert scale (1=“strongly disagree” to 5=“strongly agree”). Provides average user experience score, where higher scores reflect more favorable intervention experiences. An additional 2 items (not included in average user experience rating) assessed students’ perceptions of the duration of the intervention (1=“way too short,” 2=“too short,” 3=“just right,” 4=“too long,” and 5=“way too long”) and frequency of the reminder emails (1=“way too infrequent,” 2=“too infrequent,” 3=“just right,” 4=“too frequent,” and 5=“way too frequent”).		.93	
	User Experience Assessment part 2	8		Participants are asked to provide short-answer responses to open-ended questions about their experiences of using the app.		N/A^n^	
	Learning experiences assessment	13		Questions were adapted from Kinnunen et al [70] (items altered to suit learning objectives of this intervention). Participants rate the degree to which they agree with statements about their learning experiences using a 5-point Likert scale (1=“strongly disagree” to 5=“strongly agree”). The scale provides an average item score. Higher scores indicate higher perceived skill learning.		.90	

^a^MBI-GS(S): Maslach Burnout Inventory General Survey for Students.

^b^E: exhaustion.

^c^C: cynicism.

^d^AE: academic efficacy.

^e^MHC-SF: Mental Health Continuum-Short Form.

^f^EWB: emotional well-being.

^g^SWB: social well-being.

^h^PWB: psychological well-being.

^i^MPFI-SF: Multidimensional Psychological Flexibility Index-Short Form.

^j^PF: Psychological Flexibility.

^k^PI: Psychological Inflexibility.

^l^DASS-21: Depression, Anxiety, and Stress Scale-21.

^m^SUS: System Usability Scale.

^n^N/A: not applicable.

#### Burnout

Improving burnout outcomes is a core goal of this intervention, and burnout is the primary outcome for the planned efficacy trial. We assessed burnout using the gold-standard measure, the Maslach Burnout Inventory [61]. Of the range of versions available, the Maslach Burnout Inventory General Survey for Students [61] was considered the most appropriate for this cohort.

#### Well-being

Improving well-being is a core goal of this intervention, and well-being is an outcome of interest to the planned efficacy trial. We assessed this outcome using the short form of the Mental Health Continuum [19].

#### Psychological Flexibility and Inflexibility

In the planned efficacy trial, we aim to explore whether engagement with the intervention app improves medical students’ Psychological Flexibility and Inflexibility and evaluate whether changes in these processes mediate burnout and well-being outcomes [57]. As there is growing evidence that Psychological Flexibility and Inflexibility are “conceptually distinct” processes that can exert independent effects on outcomes and may respond differently to interventions [54,55], we selected the Multidimensional Psychological Flexibility Index-Short Form [54], which assesses Psychological Flexibility and Inflexibility as separate processes rather than as a single dimension [55]. This will facilitate the evaluation of potential differential intervention and mediation effects in the future efficacy study.

#### Depression, Anxiety, and Stress

In addition to burnout and well-being, the planned efficacy trial aims to explore the potential benefits of the intervention app to other psychological health outcomes relevant to medical students. As such, the Depression, Anxiety, and Stress Scale-21 [66] was administered to evaluate medical students’ psychological health outcomes more broadly.

### Feasibility Outcome Measures

#### Behavioral Use Data

##### App Functionality

The app collected participants’ use data, including which assessment and intervention components were accessed and in which order. This allowed us to observe whether the app facilitated participants’ progress through the study protocol in the intended way. We defined the app as *functional* if the outcome measures and intervention components were delivered to all participants at the intended time and in the intended order, if individualization procedures were implemented as intended, if at least one participant completed the study protocol in its entirety, and if the app generated data output required to evaluate intervention outcomes.

##### Engagement

Engagement at key points in the study was defined as the number of participants who (1) downloaded the app, (2) completed all outcome measures at *t*_0_, (3) completed the introductory module at stage 1, (4) completed all outcome measures at *t*_1_, (5) completed at least one skill training activity at stage 2, (6) completed all outcome measures at *t*_2_, and (7) were still using the app at least 2 weeks after their initial sign-up.

##### Frequency of App Use

Frequency of app use was defined as the number of log-ins during the study and the number of skill activities accessed and completed.

##### Time of App Use

The app captured the time of day of each log-in, which we categorized using the following definitions: *early morning* (5 AM to 9 AM), *morning* (9 AM to noon), *afternoon* (noon to 5 PM), *evening* (5 PM to 9 PM), *late night* (9 PM to midnight), and *after midnight* (midnight to 5 AM).

#### Self-report Measures of Usability and User Experiences

Self-report usability and user experience measures (Table 2) were administered at *t*_1_ (to obtain students’ feedback on the intervention following the stage 1 introductory module) and *t*_2_ (to obtain feedback following the stage 2 individualized skill training access period).

##### App Interface Usability

We assessed medical students’ satisfaction with the usability of the app as subjective perceptions of the functionality of a digital psychological intervention can influence engagement [26,71]. The System Usability Scale (SUS) is a valid and reliable measure of this outcome [72] and is the most frequently used subjective measure of program usability [71].

##### User Experiences

We used the theoretical framework of acceptability (TFA) [31] to guide the assessment of user experiences with the intervention. The TFA is a comprehensive research-based model that recommends evaluating participant-rated acceptability of health care interventions using 7 emotional and cognitive indicators: affective attitude, perceived effectiveness, intervention coherence, self-efficacy, opportunity costs, burden, and ethicality [31]. As there were no available standardized TFA measures, we used the definitions of the 7 indicators to inform the development of a 2-part User Experience Assessment for the purpose of this study (see Multimedia Appendix 5 for indicator definitions and assessment questions). The development of the questions was further guided by research related to factors that affect the acceptability of psychological interventions among medical students (eg, time [28], privacy [6,47], and accessibility and flexibility [28]), health care interventions (eg, affective experiences and satisfaction [31]), and digital interventions (eg, self-guided [26], expectation of benefit [73], quality, and content relevance [26]). Where appropriate, questions were framed with respect to the outcomes targeted by the intervention (ie, burnout and well-being) to evaluate factors such as the perceived effectiveness and relevance of this approach among medical students. Part 1 asked participants to indicate their level of agreement with statements about their experiences of using the Psychological Flexibility app. We defined items with an average score of <3.8 as aspects of the intervention that might require revision before the planned efficacy trial. This value was selected as it represented an average rating of a little below *agree*. We defined items with an average score of >4 (“agree”) as intervention strengths. Part 2 consisted of 8 short-answer questions, which were included to collect feedback regarding facilitators of and barriers to engagement as well as other information that we may not have considered.

##### Learning Experiences

As part of assessing the feasibility of adapting ACTraining into a digital learning format, we identified key learning goals and asked medical students to rate the degree to which they felt that these had been achieved by engaging with the app. A learning experiences assessment previously used in a similar web-based intervention study [70] was adapted to align with the focus of this app (Multimedia Appendix 6 [70]). Questions related to medical students’ self-reported ability to identify Psychological Inflexibility processes and apply Psychological Flexibility skills across each of the core processes.

##### Skill Activity Feedback

After completing a skill activity during stage 2, participants were asked to rate whether they liked the activity using a single-item measure assessed on a binary scale (using a thumbs-up or thumbs-down icon). This method of evaluating user experiences of intervention content has been used in previous digital intervention studies [74].

##### Intervention Harms

Participants were invited to report concerns or harms experienced during the study using contact links provided within the app and via email communications sent during the trial (eg, daily reminders).

### Data Analyses

#### Quantitative Data

Descriptive analyses (including mean, SD, mode, minimum, and maximum) of quantitative demographic, psychological, feasibility, and behavioral engagement outcome data were performed using the Jamovi statistical software (version 2.3.0) [75].

#### Qualitative Data

Participants’ short-answer responses to the open-ended user experience questions were evaluated qualitatively. Responses were independently reviewed by 2 members of the research team (ED and BK) and coded thematically according to their relevance to usability, accessibility, external factors (eg, time), behavioral factors (eg, practice), or any of the TFA indicators (affective attitude, perceived effectiveness, intervention coherence, self-efficacy, opportunity costs, burden, and ethicality). Each response was coded as a barrier to or facilitator of engagement or a positive or negative evaluation of an aspect of the intervention. The coding was then reviewed together to determine common themes and subthemes in a reiterative process.

## Results

### Participant Characteristics at Baseline (*t*_0_)

#### Demographics

Figure 1 shows that 19 medical students enrolled and 11 (58%) actively engaged in the study and provided user experience feedback. Of the 11 actively engaged participants, 10 (91%) identified as female, 10 (91%) were enrolled as domestic students, and 2 (18%) were Aboriginal individuals. Participants were aged between 20 and 23 (mean 21, SD 1.2) years. The average time engaged in the workforce was 4.5 years (SD 2.3), and medicine was the first career for most (10/11, 91%) participants. In total, 73% (8/11) of the participants reported having experienced burnout in the past. Actively engaged participants rated their physical health as *good* (mean 3.8, SD 0.4). Diet (mean 3.6, SD 0.5) and self-care (mean 3.5, SD 0.7) ratings fell between *average* and *good*.

Of the participants lost to follow-up, 50% (4/8) identified as male, reflecting that most of the enrolled male students (4/5, 80%) discontinued the study before engaging in or providing feedback on the intervention. Although the demographic characteristics of the students lost to follow-up were generally similar to those of the students who engaged in the intervention, we noted that reported self-care was lower for students who discontinued early (mean 2.8, SD 0.7).

#### Psychological Characteristics

##### Summary

Table 3 shows baseline psychological characteristics of the participants who actively engaged in the study.

**Table 3:** Baseline psychological characteristics of actively engaged participants (N=11).

Outcome		Mean (SD)	Range
**Burnout**			
	Emotional exhaustion	16.00 (6.24)	7-24
	Cynicism	8.45 (3.50)	5-16
	Academic efficacy	24.82 (5.90)	18-32
**Well-being**			
	Emotional	11.36 (2.54)	6-14
	Social	13.45 (4.06)	8-20
	Psychological	19.55 (4.57)	14-27
	Psychological Flexibility	3.71 (0.67)	2.67-5
	Psychological Inflexibility	3.03 (0.47)	2.25-3.92
	Depression	8.55 (6.82)	0-20
	Anxiety	8.18 (7.12)	0-22
	Stress	14.4 (10.23)	0-32

##### Burnout

Mean total scores for exhaustion (mean 16.00, SD 6.24) and cynicism (mean 8.45, SD 3.50) for actively engaged students were comparable with those of a recently published sample of medical students who, by subjective self-report, were not experiencing burnout (exhaustion mean 14.96; cynicism mean 7.59) [63]. Conversely, our sample’s mean total score for academic efficacy (mean 24.82, SD 5.90) was comparable with that of a sample of medical students who believed themselves to be experiencing burnout (mean 24.81) [63].

##### Well-being

On average, participants’ emotional (mean 11.36, SD 2.54), social (mean 13.45, SD 4.06), and psychological (mean 19.55, SD 4.57) well-being scores were slightly lower than those of a comparable published sample of medical students (emotional well-being mean 12.79; social well-being mean 14.84; psychological well-being mean 24.42) [76].

##### Psychological Flexibility and Inflexibility

Multidimensional Psychological Flexibility Index-Short Form scores indicated that, on average, participants had had experiences consistent with Psychological Flexibility *often* to *very often* during the previous 2 weeks (mean 3.71, SD 0.67), which was comparable with a general population sample (mean 3.83) [77]. On average, students reported that they had *often* had experiences consistent with Psychological Inflexibility during the previous 2 weeks (mean 3.03, SD 0.47), which was slightly more frequent than the general population sample (mean 2.73) [77].

##### Depression, Anxiety, and Stress

The average Depression, Anxiety, and Stress Scale-21 scores indicated that medical students in this trial were experiencing depression (mean 8.55, SD 6.82), anxiety (mean 8.18, SD 7.12), and stress (mean 10.36, SD 10.23) at levels higher than the general population averages (depression: mean 6.34, SD 6.97; anxiety: mean 4.7, SD 4.91; stress: mean 10.11, SD 7.91) [66].

### Feasibility Outcomes

#### Behavioral Use Data

##### App Functionality

The app generally functioned as intended during the trial. Use data demonstrated that outcome assessments and intervention components were delivered in the intended order and at the intended time, individualization procedures were implemented as planned, 16% (3/19) of the participants successfully completed the intervention protocol in its entirety, and the app generated the data output required to evaluate intervention outcomes. We noted that a system glitch resulted in participants being sent the daily reminder email up to 4 times each day.

##### Engagement

Of the 19 participants who were enrolled in the study, 15 (79%) downloaded the app and created an account, 14 (74%) completed all *t*_0_ outcome measures, 11 (58%) completed stage 1 and the *t*_1_ outcome measures, 8 (42%) engaged in stage 2, and 3 (16%) completed the *t*_2_ outcome measures (Figure 1). Of the participants who downloaded the app and created an account, 47% (7/15) continued to use the app for at least 2 weeks from the date of their initial log-in.

##### Frequency of App Use

Students who were actively engaged in the study (11/19, 58%) logged into the app between 2 and 10 times (total=70; mean 6.36, SD 2). Log-in frequency diminished between stage 1 (total=41; mean 3.73, SD 1) and stage 2 (total=29; mean 2.64, SD 2). It took students between 1 and 5 sittings to complete all the stage 1 activities. Participants who logged in during stage 2 (8/11, 73%) accessed a total of 24 skill activities (mean 2.18, SD 1), of which 16 (67%) were completed (mean 1.45, SD 1).

##### Time of App Use

Table 4 shows that 56% (39/70) of app use was distributed fairly evenly throughout the day (between 9 AM and 9 PM) and that medical students used the app most frequently between 9 PM and midnight (21/70, 30%). Although reasonably infrequent (5/70, 7%), medical students occasionally used the app after midnight.

**Table 4:** Frequency of app use by time of day (percentage of total log-ins; N=70).

Time of log-in	Log-in frequency, n (%)
Early morning (5 AM to 9 AM)	5 (7)
Morning (9 AM to noon)	12 (17)
Afternoon (noon to 5 PM)	14 (20)
Evening (5 PM to 9 PM)	13 (19)
Late night (9 PM to midnight)	21 (30)
After midnight (midnight to 6 AM)	5 (7)

#### Self-report Data

##### Overview

At *t*_1_, self-report measures of user experiences were completed by 100% (11/11) of the participants who completed stage 1. At *t*_2_, these measures were completed by 38% (3/8) of the participants who engaged in stage 2. Owing to the low response rate at *t*_2_, we report the minimum and maximum scores for each outcome instead of the average scores.

##### App Interface Usability

The average total SUS score at *t*_1_ (mean 85.0, SD 9.3) indicates excellent usability [68]. Individual participant SUS scores ranged between 70 (low marginal) and 96 (excellent), indicating that there were substantial differences in participants’ satisfaction with the usability of the app interface. Similarly, at *t*_2_, individual usability ratings varied, ranging between 72 and 96.

The qualitative feedback provided by participants at *t*_1_ offers insights into the factors that may have influenced this variability. Many students reported feeling satisfied by the ease of use and navigation, logical structure and flow of the app interface, provision of simple and clear instructions, and continuation of audio play when their device auto-locked. However, app usability elements that generated dissatisfaction included lack of access to a dashboard or home screen during stage 1, insufficient feedback regarding how far students had progressed through the introductory module, having to complete module components in a fixed order rather than being able to navigate flexibly through the concepts, and the delivery of reminders via email rather than in-app *push* notifications. Although some students favored the audio delivery of content, others reported that having a written version of all content would have improved accessibility (eg, audio content was a barrier to usability when in public, and audio content delivery did not match personal learning styles for some).

##### User Experiences

At *t*_1_, the average total user experience ratings ranged between *slightly agree* (3.4) and *strongly agree* (4.8; mean 4.05, SD 0.34), indicating that participants who engaged in stage 1 generally found the intervention to be acceptable with respect to the experiences assessed. Similar findings were observed at *t*_2_ (range 3.3-4.6).

The average participant scores for each user experience question at *t*_1_ are presented in Multimedia Appendix 6. We identified several intervention strengths (average item scores of >4). Strengths related to the app delivery format and interface included *quality, visual appearance, web-based availability, ability to access at a personally convenient time, maintenance of privacy,* and *did not get in the way of other important life activities.* Strengths related to the ACTraining intervention content included *opportunities for self-reflection, content relevance, anticipated benefits of continued skill use, anticipated benefits to well-being during career, concepts were easy to understand, felt confident performing the skill activities while using the app, felt capable of dealing with any challenging internal experiences that arose while using the app, aligned with personal values,* and *the time and effort invested in the Psychological Flexibility training was worthwhile.* After completing the introductory module, most students (8/11, 73%) agreed or strongly agreed that they would like to use the app again in the future (mean 3.91, SD 0.94).

We identified 5 user experience items with average scores below the 3.8 cutoff at *t*_1_, indicating that these intervention elements might require additional attention before the larger trial. These items were *enjoyed using the app, held interest and attention, received amount of training needed to achieve the goals that are important to me, felt confident using the skills in everyday life,* and *I believe these skills will help me prevent burnout.* Qualitative feedback provided by 18% (2/11) of the participants indicated that they did not feel that burnout prevention should be the focus of the intervention as the techniques were “helpful for many different things.”

Most participants (9/11, 82%) indicated that the 1-hour introductory module was *slightly too long*, whereas the remaining 18% (2/11) of participants felt that the duration was *just right*. However, qualitative feedback indicated that some participants felt that the content was as concise as it could be without compromising the value of the intervention. At both time points, participants indicated that they did not find completing the outcome questionnaires to be burdensome.

The qualitative user experience feedback at *t*_1_ generally reflected the quantitative findings. Most students who completed stage 1 provided favorable feedback about the Psychological Flexibility intervention content, describing the app as “great,” “useful,” and “helpful” and suggesting that the training “needs to be widely available.” One of the reported benefits of the app-based delivery was that students liked being able to engage in short sections and pick up where they left off rather than having to complete the entire intervention at once. Students reported enjoying the skill activities, stating that they “provided context and demonstrated how to use [the skills],” and some students requested “more interactive components and practice activities” and more “clinical examples.” *T*_1_ qualitative data also highlighted the self-efficacy challenges faced by students when accessing Psychological Flexibility skill training via an app, including feeling “too overwhelmed” in real-world situations to use the skills, not recognizing opportunities to use the skills when they arose, and not knowing which skills to apply in which situations. A participant reported feeling that they had learned the Psychological Flexibility “concepts” but not the “behavior.” These findings align with the fact that stage 1 focused on conceptual learning and participants had only engaged in minimal skill training at this stage of the intervention. Students also reported that a key barrier to using the app was having time available to do so.

Qualitative data collected at *t*_2_ provided further insights into the perceptions of a small selection of participants regarding the stage 2 individualized skill training. Students liked the app interface and the short duration of skill activities. However, the high frequency of reminders resulting from an app glitch was reported as unsatisfactory and frustrating. A participant suggested that reminders should be delivered at the end of the workday as this would align better with medical students’ schedules and reduce burden. Barriers to engagement in skill training included life commitments, time, forgetting, and the fact that the training had not yet become a priority or a habit.

##### Learning Experiences

At *t*_1_, participants’ learning experience assessment scores ranged from 2.92 (*slightly agree*) to 4.1 (*agree*; mean 3.7, SD 0.51)*.* The most frequently endorsed rating for 92% (12/13) of the learning experiences was *agree* (mode=4). At *t*_2_, scores ranged from 2.62 to 4, with a participant indicating that they did not learn the skills targeted by the intervention and 18% (2/11) of the participants agreeing that they had learned the skills.

##### Skill Activity Feedback

Students “liked” 88% (14/16) of the activities completed during stage 2.

##### Intervention Harms

No harms were reported during this trial.

## Discussion

### Feasibility, Engagement, and User Experiences

This early implementation trial with a small group of Australian medical students provided preliminary support for the feasibility of an individualized ACTraining intervention app for well-being and burnout as well as user experience feedback that may be used to guide the ongoing development of the app and future research. In relation to our first study aim, we demonstrated that the app could successfully administer outcome assessments, ACTraining intervention components, and Psychological Flexibility skill individualization procedures. These functional feasibility data were supported by subjective user experience feedback provided by medical students who actively engaged with the app. The interface was, on average, rated as having *excellent* usability, and students responded favorably to accessibility factors related to the app-based delivery (eg, maintenance of privacy and ability to access at any time). Furthermore, we demonstrated the feasibility of using an app to deliver ACTraining content and skill training to a sample of medical students in a way that they found easy to understand and acceptable.

More broadly, the feasibility of delivering psychological skill training via an app is contingent on the degree to which this method can facilitate and maintain end-user engagement with the intervention content. We found that 42% (8/19) of the medical students who enrolled in the study disengaged without completing the introductory module, and 50% (4/8) of those students did not proceed as far as downloading or signing up to the app. These students reported lower average self-care ratings than those who completed the introductory module, and future research should explore whether existing self-care behavioral patterns could impede early engagement in psychological intervention apps. Regarding maintenance of engagement, the frequency of app log-ins diminished between stages 1 and 2. Less than half (7/15, 47%) of the students who downloaded the app were still engaged after 2 weeks. Those who used the app during stage 2 completed <2 skill activities on average despite “liking” most activities completed. Although the observed pattern of disengagement over time is common for digital and app-based psychological interventions [30,78], it is incongruent with the fact that students who completed the introductory module generally reported that they would like to use the app in the future. These findings reinforce the need for a detailed formal evaluation of end-user experiences to better understand factors that may affect feasibility [1] and guide app intervention development, which was the second aim of this study.

Previous research evaluating an app-delivered ACTraining intervention indicated that disengagement is not necessarily an unfavorable outcome and might reflect the successful achievement of an individual’s learning goals [27]. User experience findings suggest that this was not the case in this sample. Learning experience scores showed that medical students who completed the introductory module agreed that they had learned most of the Psychological Flexibility skills targeted by the ACTraining app to some extent. However, on average, participants only slightly agreed that they had received the amount of training needed to achieve the outcomes that were important to them, and qualitative feedback indicated that they wanted more opportunities for skill practice. Thus, declining engagement following the introductory module was likely related to other factors.

Medical students who completed the introductory module predominantly reported finding the Psychological Flexibility concepts and skills relevant, helpful, and worthwhile. Students tended to perceive that the skills had greater benefits and relevance to well-being than burnout prevention. Given that the intervention was intended to have relevance to both outcomes, this feedback suggests that content might not have aligned sufficiently with factors that participants perceived to be relevant to their burnout experiences. A future step in the development process of this app will involve consultation with medical student end users regarding which aspects of their academic and clinical experiences they consider to be most associated with burnout risk. Intervention content can then be adjusted to demonstrate how Psychological Flexibility skills might be implemented to mitigate these risk factors, which could increase perceived relevance and engagement. Some students astutely recognized the potential for Psychological Flexibility to offer benefits beyond burnout prevention and proposed that the intervention shift its focus and messaging to the broader relevance of these skills. This approach may increase the engagement of medical students interested in well-being and other outcomes. However, removing the burnout prevention focus could also result in failure to engage students who self-identify as being at risk as well as missing opportunities to train important risk-mitigating applications of Psychological Flexibility skills. Future research might explore whether engagement is improved by using the app to assess which outcomes are most important to individual medical students and individualizing the focus of intervention content based on these preferences.

Performing self-reflection and skill-learning tasks without in-person guidance is a potential challenge for individuals engaging in a psychological intervention using a stand-alone app [26]. Medical students who actively trialed this intervention reported both enjoyment and confidence with respect to these activities while using the app. However, lower levels of confidence and self-efficacy were reported with respect to implementing the skills in real-world situations, with some students not knowing when or how to apply specific skills and others feeling “too overwhelmed” to do so. Previous research has demonstrated that perceived self-efficacy can affect engagement in digital health interventions [73]. Although stage 2 of the app was intended to strengthen and generalize skill acquisition by providing access to targeted training in real-world contexts, clearer guidance may have been required regarding when and how to practice the skills. Future research may also consider how to use in-app assessment of students’ confidence and self-efficacy to tailor intervention delivery to their current skill level and explore whether this enhances engagement.

We identified enjoyment of the intervention and its capacity to hold medical students’ interest and attention as aspects of the app requiring further attention. Most participants who trialed the app (9/11, 82%) reported finding the 1-hour introductory module slightly too long. Given that this was very brief compared with face-to-face ACTraining interventions [79-81] and that most students reported wanting further training, reducing the volume of introductory content could affect future efficacy and is not an ideal solution. Rather, qualitative user experience feedback highlighted aspects of the app interface that may have contributed to this module feeling subjectively long. Delivery of content in a fixed order was a frustration for many students, who reported finding it difficult to track how far they had progressed through the module and would have preferred the autonomy to move between topics using a home screen. Students indicated that they would have liked more interactive components and opportunities to practice skills and self-reflection, suggesting that the introductory content could have been delivered in a more engaging format. Furthermore, although many students responded favorably to the audio-delivered content, others reported that the availability of a written version would have increased accessibility and engagement. Medical students’ preferences for interacting with app-based media appeared to affect their experiences of participating in the intervention. When adapting established psychological intervention models to app-based intervention models, future research may explore whether evaluating and accommodating medical students’ interface-related preferences strengthens interest and engagement.

Feedback provided by medical students who actively participated in the intervention indicated that the short and resumable nature of the app-delivered skill activities facilitated engagement and that the time invested was worthwhile. However, time factors and demands related to medical studies were reported barriers to app use, which is consistent with previous research evaluating an app-delivered psychological intervention for medical residents [22]. Participant feedback suggested that this might be mitigated by amending the format and timing of reminders, such as *push* notifications at the end of the day, when there are fewer competing demands associated with medical studies. This approach is supported by the observed timing of app engagement occurring frequently after 9 PM. Although this intervention focused on strategies to support well-being behaviors in the context of time pressures and demands, future interventions should introduce these components during the early stages of training, when app engagement is at its highest.

Medical students in this sample reported depression, anxiety, and stress experiences at levels higher than those observed in general population samples [66], and previous research indicates that these factors can affect engagement in digital interventions [73]. Furthermore, this sample reported high baseline Psychological Inflexibility compared with a general population sample [77]. Although the ACTraining app was intended to improve this outcome, if existing inflexible behavioral repertoires function as barriers to engagement, then steps would need to be taken to mitigate this during the early stages of the intervention. Future research should explore whether baseline flexibility and inflexibility predict app engagement.

### Limitations

Although this trial had the full support of the universities in which it was conducted, the intervention was intentionally distanced from students’ medical education programs to prevent perceived participation coercion. Although important from a research ethics perspective, this meant that the app was delivered in isolation without external contextual factors that might have facilitated engagement. For example, delivery as a formal part of medical education training could normalize and legitimize the value of well-being skill training, which may strengthen students’ justification for prioritizing and allocating time for engagement [5,6]. There is support for individual skill training interventions to be formally incorporated into broader well-being and burnout prevention initiatives within medical education [10], and future research should explore whether this approach could strengthen engagement in app-based training.

Engagement rates for this trial should be interpreted with caution. The volume of study-related outcome measures that were extraneous to the intervention itself was high and might have contributed to disengagement, particularly given that time was reported as a barrier to app engagement even among those who participated for the full duration of the study. Although medical students who completed the outcome measures did not find them to be burdensome, this may not have been the case for those who disengaged. The user experience findings were biased toward the opinions of medical students who were actively engaged in the intervention. Future feasibility studies should adopt a secondary method of follow-up data collection to assess the experiences of students who discontinue app use to facilitate a better understanding of engagement barriers.

The collection of user experience data using a nonstandardized questionnaire written for the purpose of this study was a limitation. Although the User Experience Assessment was theory-driven, it is possible that researcher subjectivity might have biased its development and response interpretation. Given the lack of quantitative scales based on the TFA [31], we have made this assessment available (Multimedia Appendix 6) to facilitate further validation and refinement of feasibility assessment strategies for psychological app development studies.

The skew toward female participants and small sample size prevent the generalizability of the observed findings to medical students more broadly. Furthermore, although subjective feedback was informative regarding what was important to participants while using the app, we were unable to confirm whether these factors affected actual behavioral engagement because of the sample size and study design, and further research is required to explore the proposed relationships. Finally, the low level of engagement with the stage 2 individualized skill training component meant that most of the user experience data collected pertained to the introductory module only.

### Theoretical Contributions and Conclusions

Despite its limitations, this study reports detailed user experience data that have been largely absent from the literature on digital psychological interventions for medical students [1] and outlines a process for the important step of assessing feasibility outcomes as part of app development. The findings support the feasibility of implementing this app in a larger efficacy trial and provide user experience feedback that could be used to improve the intervention and strengthen engagement. Intervention development was driven by research suggesting the relevance of Psychological Flexibility skills to medical students with respect to burnout and well-being [6,47-50] and the potential benefits of app-delivered psychological skill training for this group [1,6,12,26-29]. Although this is early-phase work, the study provides preliminary support for the feasibility of delivering an ACTraining intervention to medical students using a stand-alone app and supports the value of further evaluation of this approach in burnout prevention and well-being research. Psychological Flexibility has recently been described as “the cornerstone of psychological health and resiliency” [82] because of its broad well-being benefits. With continued evaluation and refinement, ACTraining apps offer a promising approach to facilitating medical students’ development of these important skills, which could be useful in the context of unavoidable academic and occupational stressors.

This study is the first to report detailed feedback from medical student end users regarding their experience of engaging in an app-delivered psychological intervention. Although specific findings may not be generalizable beyond this sample and app, our observations and evaluation process have potential relevance to researchers in the emerging field of app-delivered psychological interventions for medical student well-being. We demonstrated that making an intervention accessible to medical students via an app does not guarantee that they will access it, even when their self-reported intention is to do so, and that formal evaluation of user experiences across a range of domains may facilitate the identification of contributing factors. Our findings suggest that it may be useful for intervention developers to assess medical students’ preferences regarding the functionality and usability aspects of app interfaces as well as individual differences in learning goals, baseline skills, and confidence. Failure to understand user experiences could result in disengagement for reasons unrelated to the intervention model, leading to missed opportunities to fully develop and deploy potentially valuable psychological skill training [1]. We recommend that future researchers incorporate formal evaluation of medical students’ subjective user experiences into the intervention development process (including quantitative and qualitative assessments) to better understand and address factors that might affect engagement and disengagement.

## Appendix

Multimedia Appendix 1 Stage 1 screenshot.

Multimedia Appendix 2 App home screen.

Multimedia Appendix 3 App screening questions.

Multimedia Appendix 4 Stage 2 skill set dashboard.

Multimedia Appendix 5 Acceptability assessment and mean participant data at *t*_1_ (n=11).

Multimedia Appendix 6 Learning experiences assessment.

## Abbreviations

ACTraining: Acceptance and Commitment Training
REDCap: Research Electronic Data Capture
SUS: System Usability Scale
TFA: theoretical framework of acceptability

## Data Availability

The data sets generated and analyzed during this study are not publicly available for the protection of participant privacy, but deidentified outcome data sets are available from the corresponding author upon reasonable request.
