# Supplementary figures and images for: Medical Student Experiences of Engaging in a Psychological Flexibility Skill Training App for Burnout and Well-being: Pilot Feasibility Study
Source: JMIR Form Res. 2023 Jan 10;7:e43263. doi: 10.2196/43263 (PMC9874998; doi:10.2196/43263)

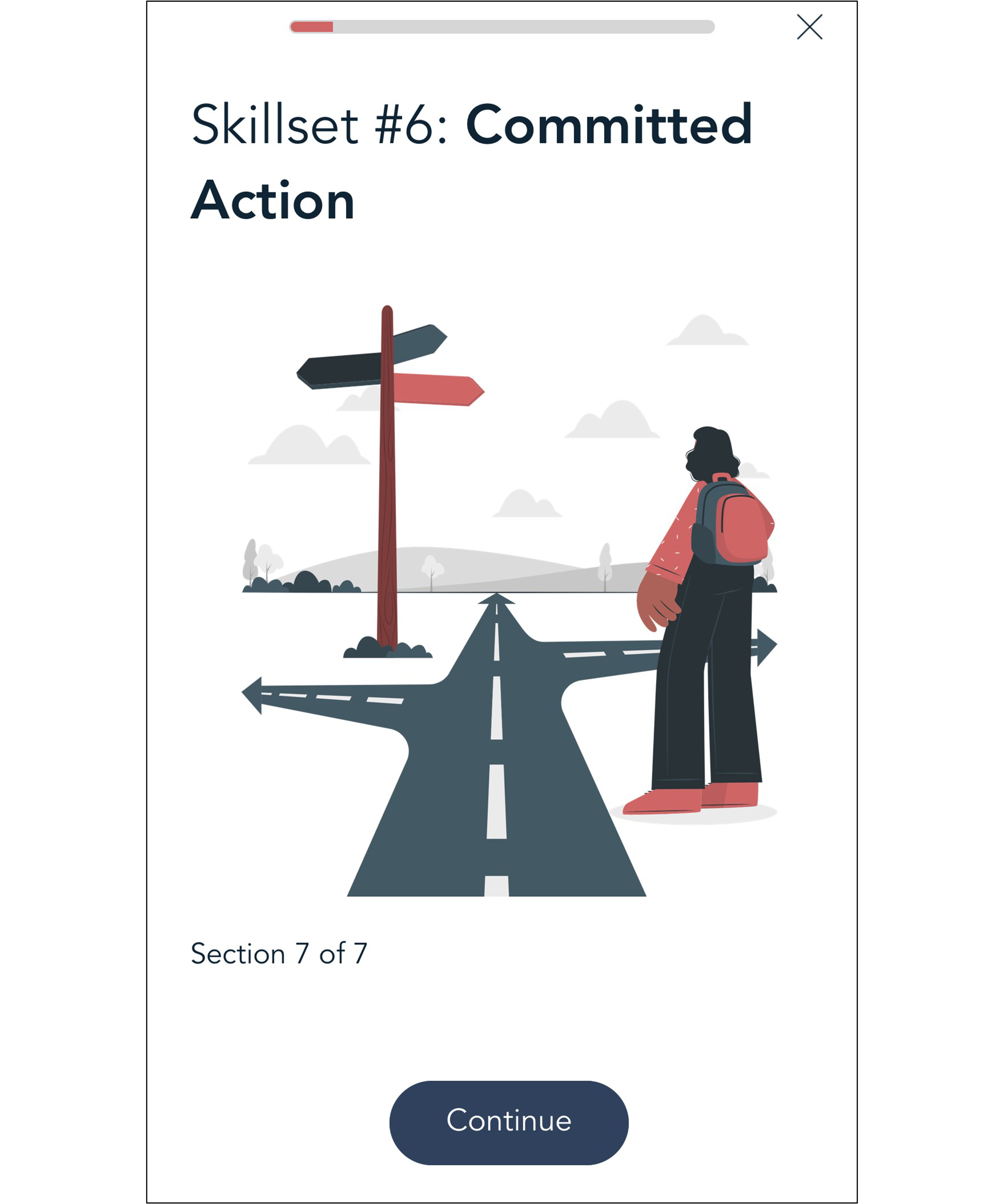

Supplement: Multimedia Appendix 1 [file formative_v7i1e43263_app1.png]

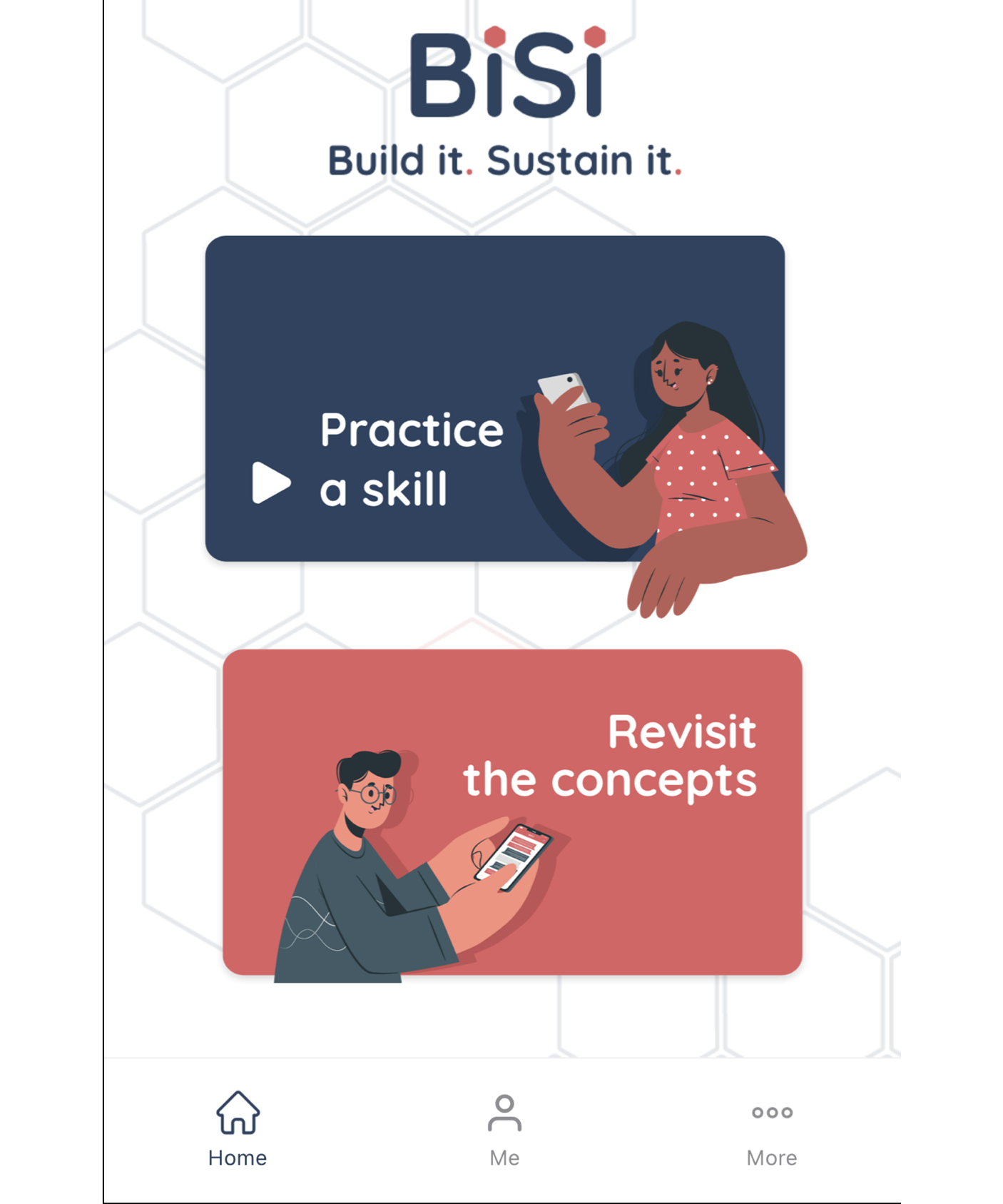

Supplement: Multimedia Appendix 2 [file formative_v7i1e43263_app2.png]

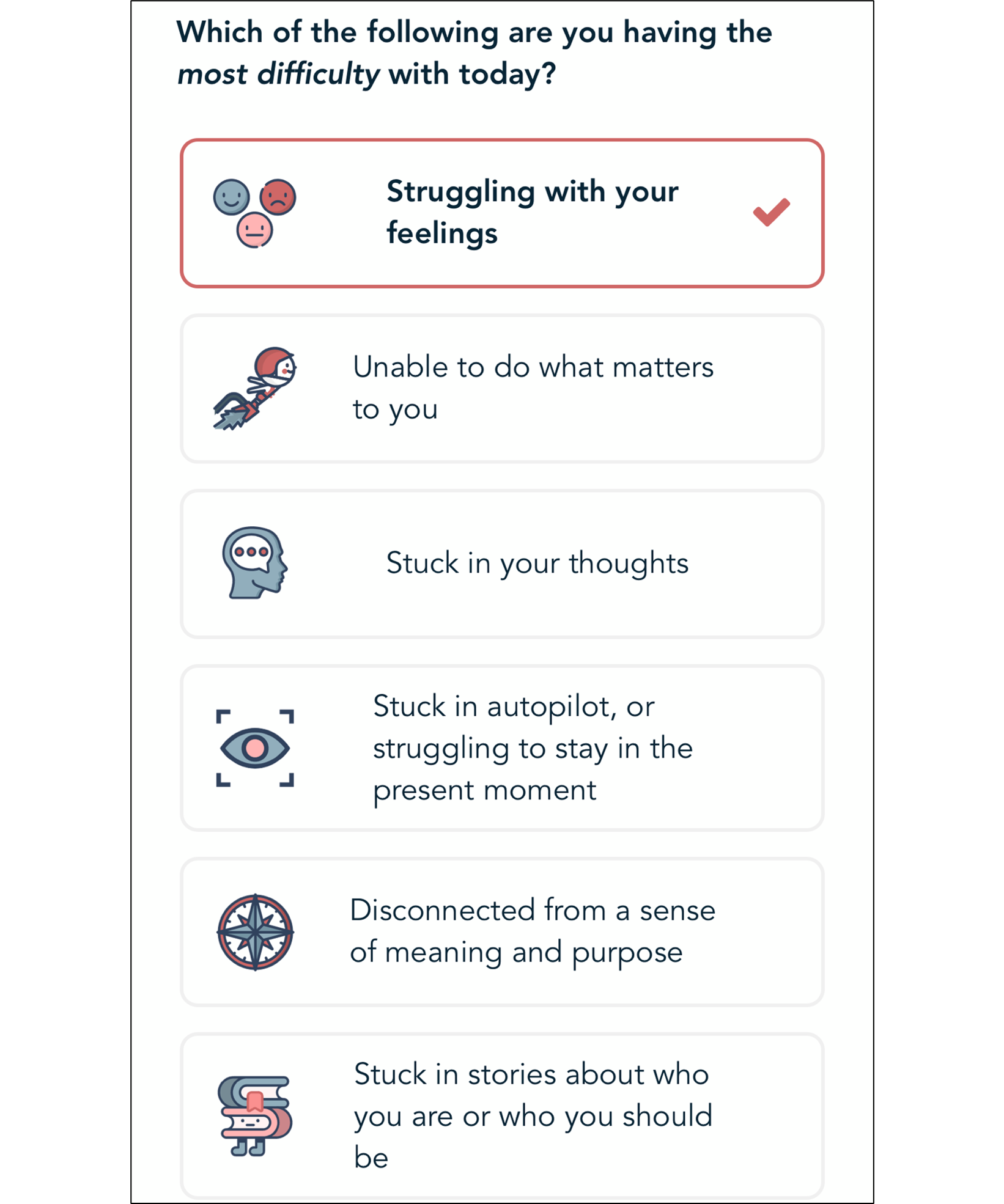

Supplement: Multimedia Appendix 3 [file formative_v7i1e43263_app3.png]

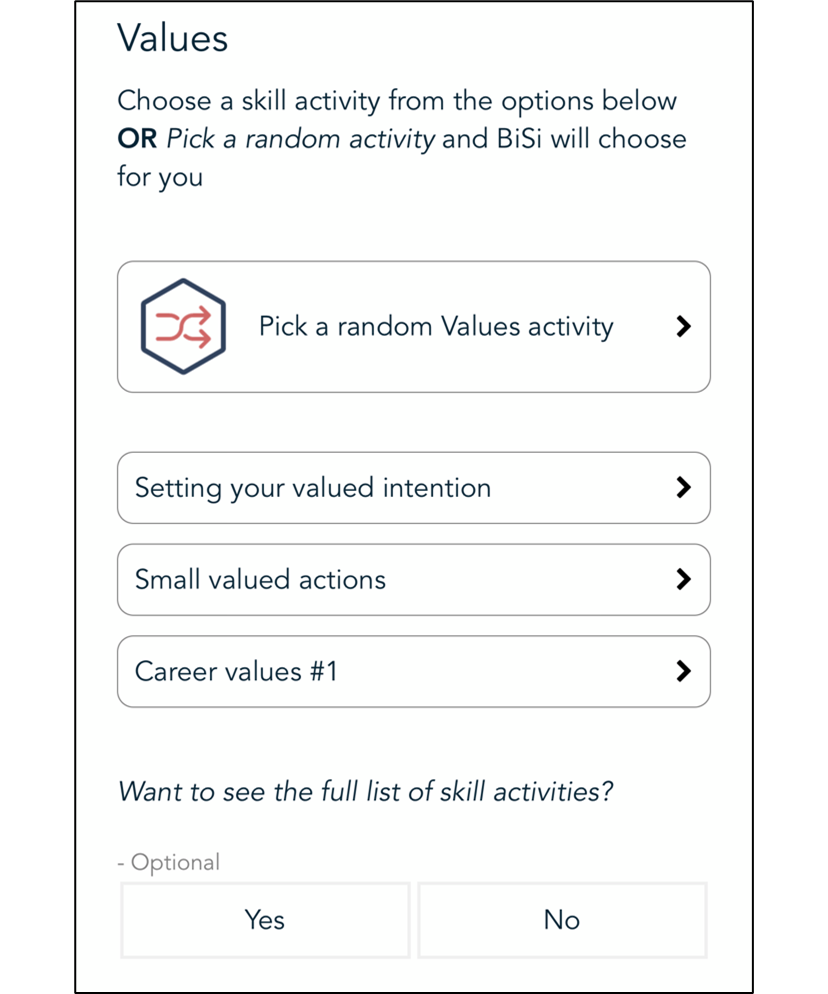

Supplement: Multimedia Appendix 4 [file formative_v7i1e43263_app4.png]
