# Supplementary material for: Medical Student Experiences of Engaging in a Psychological Flexibility Skill Training App for Burnout and Well-being: Pilot Feasibility Study
Source: JMIR Form Res. 2023 Jan 10;7:e43263. doi: 10.2196/43263 (PMC9874998; doi:10.2196/43263)
Supplement: Multimedia Appendix 5 [file formative_v7i1e43263_app5.docx]

**Appendix 5: User Experience Assessment (based on TFA definitions (Sekhon et al., 2018)) and Mean participant data at T_1_ (n=11)**

Part 1:

|  | Item | Mean (SD) | Mode | Min-Max |
| --- | --- | --- | --- | --- |
| Affective Attitude: The way participants felt about various aspects of the intervention | | | | |
| 1 | I enjoyed using the App ***** | 3.73 (0.65) | 4 | 2 - 4 |
| 2 | I was satisfied with overall quality of App | 4.00 (1.00) | 4 | 2 - 5 |
| 3 | The app held my interest and attention ***** | 3.55 (0.82) | 4 | 2 - 5 |
| 4 | I liked the visual appearance of App | 4.18 (0.75) | 4 | 3 - 5 |
| 5 | I would like to use this App again in the future | 3.91 (0.94) | 4 | 2 - 5 |
| 6 | I would recommend this App to others | 3.82 (0.87) | 4 | 2 - 5 |
| 7 | I liked the online delivery format | 4.09 (0.70) | 4 | 3 - 5 |
| 8 | I liked being able to access the training at a time that suited me | 4.64 (0.50) | 5 | 4 - 5 |
| 9 | The App allowed me to maintain my privacy | 4.18 (0.60) | 4 | 3 - 5 |
| 10 | I liked engaging in self-reflection while using the App | 4.18 (0.75) | 4 | 3 - 5 |
| Perceived Effectiveness: Participants’ perceptions of intervention benefits | | | | |
| 11 | The App was helpful | 3.82 (0.75) | 4 | 3 - 5 |
| 12 | The activities were relevant to my needs | 4.00 (0.63) | 4 | 3 - 5 |
| 13 | I received the amount of training needed to achieve outcomes important to me ***** | 3.09 (1.14) | 3 | 1 - 5 |
| 14 | I think that continuing to practice these skills will be helpful to me | 4.45 (0.52) | 4 | 4 - 5 |
| 15 | I believe that the skills I have learned in this App will help support my wellbeing during my career | 4.00 (0.45) | 4 | 3 - 5 |
| 16 | I believe that the skills I have learned in this App will help me prevent burnout ***** | 3.55 (0.82) | 4 | 2 - 5 |
| 17 | Self-reflection was helpful while using the App | 4.00 (0.63) | 4 | 3 - 5 |
| 18 | Self-reflection will help with my future responses to challenging experiences | 3.82 (0.75) | 4 | 3 - 5 |
| 19 | Self-reflection helped me clarify what I can do to improve my wellbeing in the future | 3.82 (0.98) | 4 | 2 - 5 |
| Intervention Coherence: How well participants were able to comprehend the intervention and how it worked | | | | |
| 20 | It was clear to me how the App activities and skills were relevant to my experiences | 4.00 (0.89) | 4 | 2 - 5 |
| 21 | It was clear to me how the App activities and skills were relevant to my goals | 4.27 (0.65) | 4 | 3 - 5 |
| 22 | It was clear to me how the App activities and skills were relevant to my wellbeing | 4.36 (0.50) | 4 | 4 - 5 |
| 23 | It was clear to me how the App activities and skills were relevant to my risk of burning out | 3.82 (0.87) | 4 | 2 - 5 |
| 24 | The concepts were presented in a way that I could understand them | 4.73 (0.47) | 5 | 4 - 5 |
| 25 | It was clear to me why self-reflection while using the App was important | 4.36 (0.50) | 4 | 4 - 5 |
| Self-Efficacy: The degree to which participants believed they possessed the behavioural skills to engage in the intervention activities | | | | |
| 26 | I felt capable of performing the skills and activities taught during the App | 4.09 (0.70) | 4 | 3 - 5 |
| 27 | I felt confident using the skills learnt in this App in everyday life ***** | 3.73 (0.90) | 4 | 2 - 5 |
| 28 | I felt able to handle the challenging experiences, thoughts, emotions, behaviours and/or physical sensations I was asked to reflect on while using the App | 4.18 (0.60) | 4 | 3 - 5 |
| Opportunity Costs: How much participants perceived their involvement in the intervention cost them with respect to other important aspects of their lives | | | | |
| 29 | The time and effort taken to participate in this study was worthwhile | 4.09 (0.83) | 5 | 3 - 5 |
| 30 | Using the App got in the way of other important things in my life (reverse-scored) | 4.55 (0.52) | 5 | 4 - 5 |
| Ethicality: The degree of congruence between participants’ personal value systems and those of the intervention | | | | |
| 31 | The intervention aligned with my values | 4.27 (0.47) | 4 | 4 - 5 |
| Burden: Participants’ perceptions of any adverse time and effort impacts of their engagement in the intervention | | | | |
| 32 | The time required to complete questionnaires was burdensome (reverse-scored) | 4.18 (0.75) | 4 | 3 - 5 |

***** Intervention components identified as requiring additional attention prior to future efficacy trial (M<3.8)

Part 2 Questions:

1. What got in the way of you using the App?
2. What made it easy to use the App?
3. What got in the way of you using these skills in your everyday life?
4. What made it easier for you to use these skills in your everyday life?
5. What changes could be made to the App to make it more *accessible* to you and others in your course?
6. What changes could be made to the App to make it more *relevant* to you and others in your course?
7. What changes could be made to the App to make it more *interesting and enjoyable to use*?
8. Is there any other feedback you would like to provide to help us improve this App?
