# Supplementary material for: Medical Student Experiences of Engaging in a Psychological Flexibility Skill Training App for Burnout and Well-being: Pilot Feasibility Study
Source: JMIR Form Res. 2023 Jan 10;7:e43263. doi: 10.2196/43263 (PMC9874998; doi:10.2196/43263)
Supplement: Multimedia Appendix 6 [file formative_v7i1e43263_app6.docx]

**Appendix 6 – Learning Experiences Assessment**

When answering these questions, think about what you have learned from this App.

| Strongly Disagree | Disagree | Slightly Agree | Agree | Strongly Agree |
| --- | --- | --- | --- | --- |

1. I have learned to be aware of my thoughts, emotions, and bodily reactions
2. I have learned to recognise my behaviour patterns and how they relate to important outcomes in my life
3. I have learned to “buy out” of unhelpful thought processes
4. I have learned to notice when I am “stuck” in my thoughts
5. I have learned how my stories about myself or self-labels can limit the choices available to me in a challenging situation
6. I have learned to apply awareness skills in my daily life
7. I have learned to notice my automatic reactions
8. I have learned to adopt an attitude of curiosity to my thoughts, emotions, and physical sensations
9. I have learned to allow my uncomfortable internal experiences to come and go without struggling against them
10. I have learned to clarify my values
11. I have learned to plan values-based actions
12. I have learned to define what I can do to promote my wellbeing in relation to my work/study
13. I have learned to view my experiences from a broader perspective

***Adapted from*** *Kinnunen, S. M., Puolakanaho, A., Tolvanen, A., Mäkikangas, A., & Lappalainen, R. (2019). Does mindfulness-, acceptance-, and value-based intervention alleviate burnout?—A person-centered approach. International Journal of Stress Management, 26(1), 89-101. doi:10.1037/str0000095*
